# Regulation of Transketolase Like 1 Gene Expression in the Murine One-Cell Stage Embryos

**DOI:** 10.1371/journal.pone.0082087

**Published:** 2014-01-02

**Authors:** Go Hamamoto, Tsukasa Suzuki, Masataka G. Suzuki, Fugaku Aoki

**Affiliations:** Department of Integrated Biosciences, Graduate School of Frontier Sciences, University of Tokyo, Kashiwa, Chiba, Japan; Baylor College of Medicine, United States of America

## Abstract

In mice, transcription from the zygotic genome starts at the mid-one-cell stage after fertilization. Previous studies showed that an enhancer is not required for transcription at this stage, and that the enhancer-dependent mechanism of transcription is established during the two-cell stage. However, these results were obtained using reporter gene assays with promoters derived from viruses, rather than from endogenous genes. We conducted a reporter-gene assay using the promoter of *Tktl1*, which is transcribed after fertilization, to investigate the mechanism regulating gene expression at the one-cell stage. When a plasmid containing the 2467 bp upstream and 25 bp downstream of the *Tktl1* transcription start site (TSS) was microinjected into the nuclei of growing oocytes, and one-cell stage and early and late two-cell-stage embryos, transcriptional activity was detected in the one-cell- and two-cell-stage embryos, but not in the oocytes. It was highest at the early two-cell stage and was reduced at the late two-cell stage. The decrease in activity at the late two-cell stage was prevented by inhibiting the second round of DNA replication, suggesting that the transcriptionally repressive state is established during the two-cell stage by a mechanism coupled to DNA replication. When the *Tktl1* promoter was deleted to leave 56 bp upstream of the TSS which includes GC and TATA boxes, transcriptional activity was still detected in one-cell-stage embryos, but not early or late two-cell-stage embryos. The core promoter of *Tktl1* alone seems to be able to induce basal transcription at the one-cell stage. These results suggest that repressive chromatin is established after fertilization in two steps, which occur during the transition from the one- to two-cell stage and during DNA replication at the two-cell stage.

## Introduction

In mammals, oocytes actively transcribe the genes during their growth phase and then cease transcription when they are fully grown. This transcriptionally silent state continues during meiotic maturation and even after fertilization. The first transcription from zygotic genome occurs at particular stage depending on the species. In mice, it occurs at mid-1-cell stage [1]–[3], which is followed by the major transcriptional activation at mid to late 2-cell stage [3], [4]. This zygotic transcription was so called as zygotic gene activation (ZGA), which is the first gene expression after birth of new life and shows a quite different pattern of the expressed genes from that in the oocytes [4]–[6].

To date, there were many studies by using reporter gene to elucidate the regulation of ZGA. Ram and Schultz [7] reported that the reporter gene with SV40 early prometer exhibited the transcriptional activity in the 1-cell stage embryos as well as the 2-cell ones. Wiekowski et al. [8] showed that reporter vectors with HSV tk or PyV t-ga promoter did not require the enhancer for the transcription in the 1-cell stage embryos; the vectors without an enhancer exhibited the transcriptional activity at the level comparable with those with an enhancer. However, the activity was very low without an enhancer in the 2-cell stage embryos. Thus, the embryos are in transcriptionally permissive state at 1-cell stage but become to be in repressive state that needs an enhancer for the active transcription during 2-cell stage. This transformation from transcriptionally permissive to repressive state seems to require the second round DNA replication, since the embryos were still in the permissive state at the 2-cell stage when DNA synthesis was inhibited with aphidicolin [8]–[11]. Furthermore, the treatment of the embryos with butyrate, an inhibitor of histone deacetylase had the embryos remain in the permissive state at the 2-cell stage [9], [12]. Since it has been known that hyperacetylated state caused by butyrate made the chromatin in the loosen state [13], the transcriptionally permissive state would be derived from the loosened chromatin in the 1-cell stage embryos, whereas the tight chromatin would make the repressive state at the 2-cell stage. Although these studies well documented the change in the regulation of gene expression during 1- and 2-cell stage embryos, there is some concern that the results of these studies precisely reflect the mechanism regulating the expression of endogenous genes: they used the promoters derived from virus, not the endogenous one [7]–[11]. Furthermore, the 1-cell stage embryos used in those studies were chronologically 2-cell stage ones that had been arrested at the 1-cell stage by the inhibition of the first round DNA replication by aphidicolin. It is not certain whether the gene expression in the embryos arrested at 1-cell stage is regulated in the same manner as that in the normal 1-cell stage embryos, since it is known that the inhibition of DNA synthesis by aphidicolin affects the transcriptional activity in the 1-cell stage embryos [3].

There were some studies using an endogenous gene, *Hsp70.1*. In the transgenic mice carrying the *Luc* gene with *Hsp70.1* promoter, although a very low level of transgene expression was observed only in 10% of 1-cell stage embryos, a much higher level of the expression occurred in most of early/mid 2-cell stage embryos and then it was repressed at the late 2-cell stage [14]. This repression did not occur when the 2-cell stage embryos were treated with aphidicolin. The reporter gene assay using microinjection technique revealed that in the 2-cell stage embryos, *Hsp70.1* exhibited the transcriptional activity only with the proximal promoter region where there are several regulatory elements for transcription [15], [16]. In these studies, however, the transcriptional activity of the reporter gene was examined for the 2-cell stage embryos which had been subjected to the microinjection of the reporter gene at the 1-cell stage. .

In the present study, we conducted the reporter gene assay using the promoter of an endogenously expressed gene, *transketolase like 1* (*Tktl1*) to investigate the mechanism regulating the gene expression after fertilization, especially focusing on the 1-cell stage embryos. We had previously conducted genome wide analysis for gene expression by RNA sequencing in the oocytes and preimplantation embryos and found that *Tktl1* is transcribed in the 1-cell stage embryos (Aoki, unpublished data). This is the first study that analyzed the transcriptional activity of endogenous gene promoter in the 1-cell stage embryos.

## Materials and Methods

### Collection and culture of oocytes and embryos

All procedures described here were reviewed and approved by the University of Tokyo Institutional Animal Care and Use Committee and were performed in accordance with the Guiding Principles for the Care and Use of Laboratory Animals.

Growing oocytes (GO) were collected from the ovaries of 12-day-old B6D2F1 female mice (SLC Japan, Shizuoka). Ovaries were removed and transferred into HEPES-buffered KSOM medium [28]. They were freed from the surrounding tissues with a 27G needle under the operation microscope and dissected after the treatment with trypsin to obtain the growing oocytes.

Metaphase II (MII) stage oocytes were obtained from the oviducts of 3-week-old B6D2F1 mice that had been induced superovulation by the injection of 5 IU human chorionic gonadotropin (ASKA pharmaceutical Co, Tokyo) 48 h after the administration of 5 IU of PMSG (ASKA pharmaceutical Co). For in vitro fertilization, the oocytes were transferred to human tubal fluid (HTF) medium [29] supplemented with 10 mg/ml bovine serum albumin (BSA; Sigma Aldrich) and inseminated with spermatozoa obtained from adult male ICR mouse (SLC japan, Shizuoka). The spermatozoa were pre-cultured for 2 h in HTF medium containing BSA to allow capacitation. Five h after insemination, the fertilized oocytes were washed and cultured in KSOM medium containing 3 mg/ml BSA. All incubations were done under 38°C, 5% CO_2_ and 95% air condition.

### Reverse transcription and PCR

Total RNA was isolated from oocytes and embryos using Isogen (Nippon Gene, Tokyo), and was served for reverse-transcription using a PrimeScript RT-PCR kit (Takara Bio Inc., Otsu), according to manufacturer's instructions. PCR was performed in a thermal cycler (iCycler; Bio-Rad) using Ex Taq DNA polymerase (Takara) for 35–37 cycles of 95°C for 30 s, 57°C for 30 s, and 72°C for 60 s. The primers for *Tktl1* are shown in Table 1.

**Table 1:** PCR primers.

	Primer sequence	
	Sense	Anti-sense
**<RT-PCR>**		
*Tktl1*	5′-AGGTTGTCCGCCATAGTGAC-3′	5′-GTGGCAATGTCCAGAGGTTT-3′
**<Cloning>**		
*Zp3 Upstream 480 bp*	5′-CTGCTCGAGGTCGACGA-TCCTGGTGTGGTGACATA-3′	5′-AGCACGCGTAAGCTTCT-GGGCTCAGAATGAGAGG-3′
*Tktl1Upstream 3144 bp*	5′-CTGCTCGAGGTCGACGC-TTGCAATTCCATCACTTG-3′	5′-AGCACGCGTAAGCTTTC-TACGTTGTGCCGCAGAA-3′
*Tktl1-2467+25*	5′-GGTCGACCAATGCTCAGGCCTAAAT-3′	5′-GAGCATTGGTCGACCTCGAGCAGACA-3′
*Tktl1-227+25*	5′-GGTCGACATTCATGGTGGAGGGATG-3′	5′-CCATGAATGTCGACCTCGAGCAGACA-3′
*Tktl1-56+25*	5′-GGTCGACATGACTGGGCGGGGCATG-3′	5′-CCAGTCATGTCGACCTCGAGCAGACA-3′
*Tktl1-56*	5′-GGCAGCCAAGCTTACGCGTGCTAGC-3′	5′-GTAAGCTTGGCTGCCTCAGCTGTGCC-3′
*_Tktl1-56-GCMT_*	5′-GACTGGAAAGGGCATGCCAGGGCCTTT-3′	5′-ATGCCCTTTCCAGTCATGTCGACCTCG-3′
*Tktl1-TAMT*	5′-CTGCTCGAGGTCGACAT-GACTGGGCGGGGCATG-3′	5′-AGCACGCGTAAGCTTGG- CTGCCTCAGCTGTGCC-3′

### Plasmid construction

The constructs for luciferase reporter gene assay were prepared using p*Eluc-test* plasmid which containing luciferase gene from TOYOBO (Osaka). We first constructed the plasmid harboring the −2467∼+25 bp sequence from *Tktl1* transcriptional start site (TSS) in p*Eluc*-*test* plasmid (p*Tktl1-2467+25*). A fragment of *Tktl1* promoter was amplified by PCR using genome template from a C57BL6 mouse and a primer set shown in Table 1, and cloned into multi-cloning site in p*Eluc-test* plasmid by In-fusion advantage cloning kit (Clontech, Shiga). Deletion of p*Tktl1-2467+25* was performed by using PrimeSTAR mutagenesis basal kit (Takara Bio Inc) and the primer sets shown in Table 1 to make p*Tktl1-227+25*, p*Tktl1-56+25* and p*Tktl1-56*. The p*Eluc-test* plasmid containing the 480 bp of promoter region of *Zp3* gene (p*Zp3*) was also constructed in the similar manner to p*Tktl1-2467+25*. p*Tktl1* with the mutation in GC box or TATA box was produced by site directed mutagenesis using a PrimeSTAR mutagenesis basal kit or cloning the mutated oligonucleotide to multicloning site of p*Eluc-test* plasmid with In-fusion advantage cloning kit. The GC box and TATA box was mutated from GGGCGG to GGAAAG (p*Tktl1-56-GCMT*) and TTAAAA to TTGCGA (p*Tktl1-56-TAMT*), respectively.

### Microinjection of plasmids

About 10 pl of plasmids solution adjusted to 200 ng/µl or 500 ng/µl were microinjected into the nucleus of GOs, the male pronucleus in 1-cell stage embryos at 7–9 h after insemination, and the nucleus in single blastomere of the early and late 2-cell stage embryos at 17–19 h and 26–28 h, respectively, after insemination. After the microinjection, oocytes and embryos were cultured for 6 h in α-MEM and KSOM medium, respectively, and collected in 25 µl of a phosphate buffered saline (PBS; Takara Bio Inc) containing 1 mg/ml BSA (0.1% BSA/PBS) for luciferase assay.

### Luciferase Assay

The luciferase activities of collected samples were measured with Emerald luc luciferase assay reagent (TOYOBO). The samples were added with 5 µl lysis buffer and incubated for 5 min at room temperature. After the addition with 50 µl of assay reagent, the samples were incubated for 15 min at room temperature and then transferred onto 96 wells plate and measured their luminescence by Micro Lumat plus (Berthold, Berlin, Germany). Light emission was integrated for 10 sec at 25°C in the photometer. The relative light units (RLUs) were determined by subtracting the value of none-injected sample from that of each injected samples.

### Inhibition of DNA synthesis in 2-cell stage embryos

The embryos were transferred into KSOM medium containing 3 ng/ml aphidicolin 15 h after insemination. At this time, only a small part of embryos had entered into M phase and most of embryos entered into M phase at 16 h after insemination.

## Results

### Expression of *Tktl1* during preimplantation development

**Figure 1:**
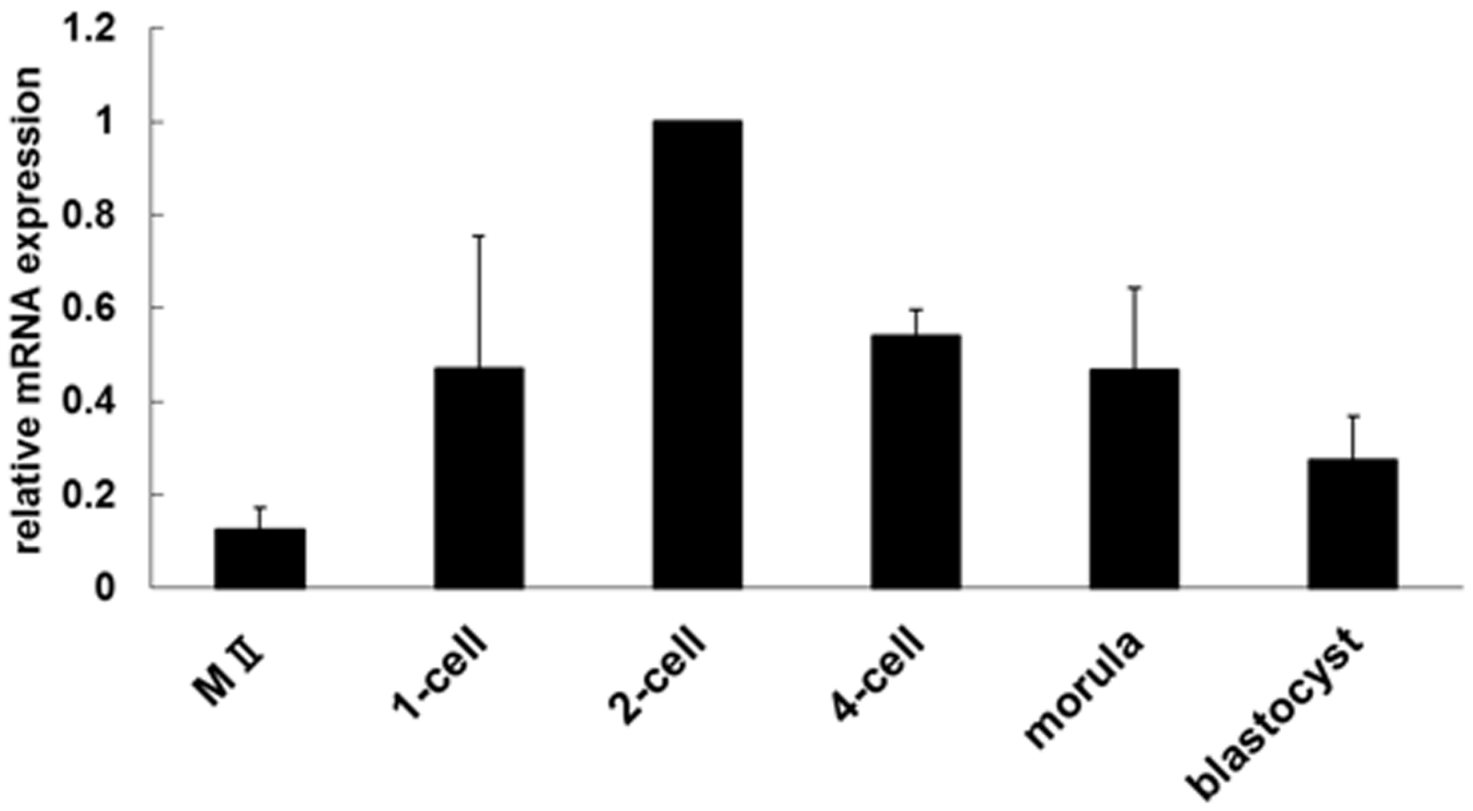
Expression of Tktl1 mRNA during preimplantation development. The expression of *Tktl1* in MII stage oocytes and preimplantation embryos was assessed by semi-quantitative RT-PCR. Embryos were collected at the following time points after insemination: one-cell stage, 13 h; two-cell stage, 28 h; 4-cell stage, 45 h; morula, 60 h; and blastocyst, 96 h. The experiments were performed four times and the data are presented as means ± SEM. The value for two-cell-stage embryos was set to 1.0 and relative values were calculated for the other stages.

The expression of *Tktl1* mRNA was examined in unfertilized oocytes and preimplantation embryos by semi-quantitative RT-PCR (Fig. 1). The expression level of *Tktl1* was low in the oocytes and increased after fertilization. It was the highest at the 2-cell stage and then gradually decreased until blastocyst stage. These results indicated that *Tktl1* is actively transcribed in the 1-cell and 2-cell stage embryos.

### *Tktl1* promoter exhibits the transcriptional activity in the 1- and 2-cell stage embryos

**Figure 2:**
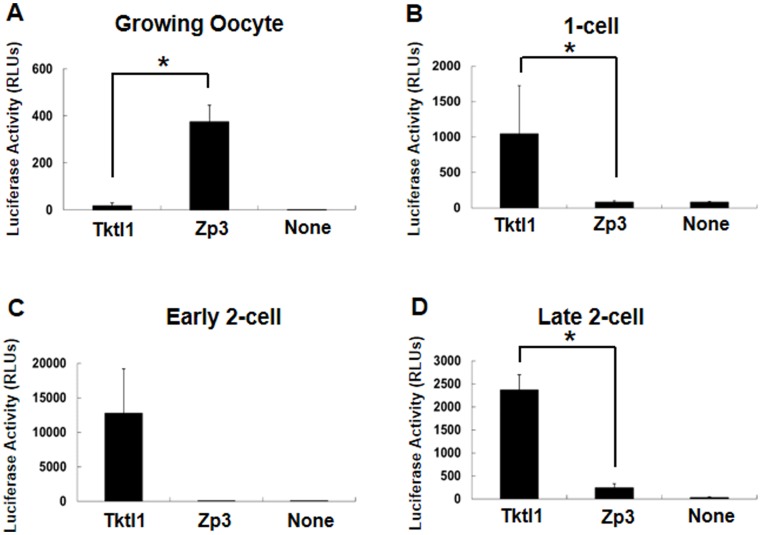
Transcriptional activity of the *Tktl1* promoter in oocytes and preimplantation embryos. Plasmids containing the promoter regions of the *Tktl1* (p*Tktl1-2467+25*) and *Zp3* genes were microinjected into growing oocytes (A), one-cell-stage embryos (B), and early and late two-cell-stage embryos (C and D), and their transcriptional activities were then evaluated by luciferase reporter gene assay. The transcriptional activity of a plasmid without a promoter (None) was used as a control to determine the background level. Plasmids (200 ng/µl) were microinjected into the nucleus. Experiments were performed at least three times and the results are presented as means ± SEM. Asterisks indicate significant differences (Student's *t*-test; *P*<0.05).

To investigate the regulation of *Tktl1* expression, we performed reporter assay with plasmid containing upstream 2467 bp and downstream 25 bp from TSS of *Tktl1* gene (p*Tktl1-2467+25*) in growing oocytes and the embryos at 1-cell and 2-cell stages. p*Tktl1-2467+25* showed transcriptional activity in the 1-cell, early and late 2-cell stage embryos but not in the oocytes (Fig. 2). It was the highest at the early 2-cell stage and reduced at the late 2-cell stage. In contrast, the plasmid containing a promoter region of *Zp3* (p*Zp3*), which is an oocyte specific gene, showed significantly higher level of transcriptional activity in the growing oocytes when compared to p*Tktl1-2467+25*, although it was much lower in the 1-cell and the late 2-cell stage embryos. The control p*Eluc* plasmid with no promoter did not show any significant transcriptional activity in either oocytes or embryos. The transcriptional activity of p*Tktl1-2467+25* was thus changed before and after fertilization in the manner consistent to the change in the expression of endogenous gene.

**Figure 3:**
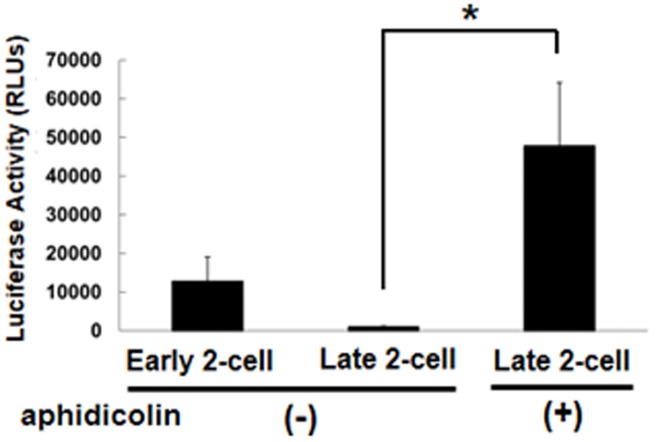
Effect of DNA synthesis inhibition on *Tktl1* transcriptional activity in two-cell-stage embryos. Transcription of p*Tktl1-2467+25* was measured in embryos in which DNA synthesis was inhibited by treatment with aphidicolin. Experiments were performed three times and the results are presented as means ± SEM. Asterisks indicate significant differences (Student's *t*-test; *P*<0.05).

It has been reported that the expression of reporter gene with the promoters derived from virus was repressed at the late 2-cell stage depending on DNA replication [8], [9]. Therefore, we examined the transcriptional activity of p*Tktl1-2467+25* under the condition that DNA synthesis was prevented by aphidicolin in the 2-cell stage embryos. The results showed that the expression of reporter gene was significantly increased by the inhibition of DNA replication (Fig. 3).

### *Tktl1* expression is regulated by basal promoter elements in the 1-cell stage embryos

**Figure 4:**
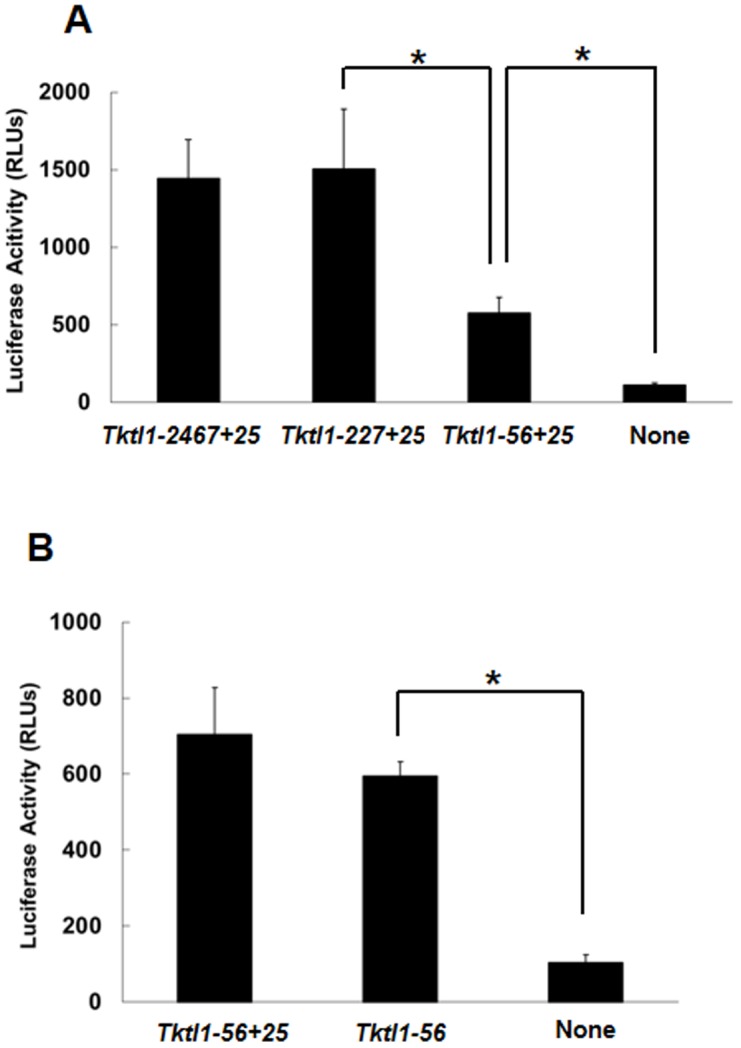
Transcriptional activity of *Tktl1*-promoter deletion mutants in one-cell-stage embryos. (A) The upstream region of the *Tktl1* promoter (*Tktl1-2467+25*) was deleted to leave 227 bp (*Tktl1-227+25*) and 56 bp (*Tktl1-56+25*), and transcriptional activity was then evaluated. (B) The 25 bp downstream of the TSS was deleted from p*Tktl1-56+25* and transcriptional activity was measured. Plasmids (500 ng/µl) were microinjected into the male pronuclei of one-cell-stage embryos. The experiments were performed at least 11 times and the results are presented as means ± SEM. Asterisks indicate significant differences (Student's *t*-test; *P*<0.05).

To find the region in p*Tktl1-2467+25* which is necessary for *Tktl1* expression in the 1-cell stage embryos, we deleted this promoter and examined for the transcriptional activity by the reporter assay. Deletion to leave only a proximal promoter region, *i.e.*, upstream 227 bp and downstream 25 bp from TSS (p*Tktl1-227+25*), did not show significant effect on the transcriptional activity (Fig. 4A). However, further deletion to leave only a core promoter region, *i.e.*, upstream 56 bp and downstream 25 bp from TSS (p*Tktl1-56+25*), significantly decreased the activity. Nevertheless, p*Tktl1-56+25* still showed ∼40% activity of p*Tktl1-2467+25*. These results suggest that transcription of *Tktl1* gene can be set off only by a core promoter and enhanced by some element(s) in a proximal promoter region in the 1-cell stage embryos. In addition, the proximal downstream region of TSS did not seem to be involved in the transcriptional activity, because deletion of the downstream 25 bp from p*Tktl1-56+25* to leave only the upstream 56 bp (p*Tktl1-56*) did not show a significant change in the activity (Fig. 4B).

**Figure 5:**
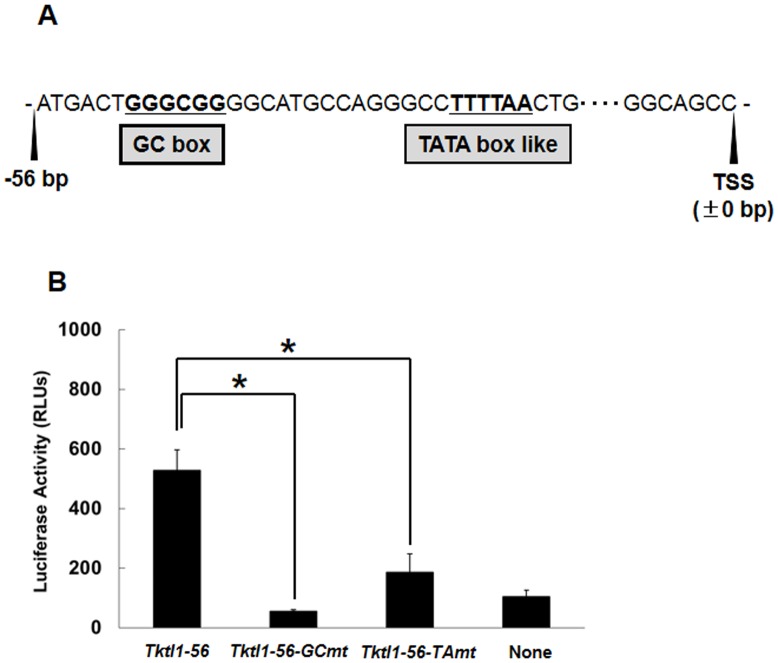
The elements required for transcriptional activity of the core promoter region of *Tktl1*. (A) Transcription-factor-binding sites in the 56 bp upstream of the TSS of *Tktl1*. (B) Effects of mutation of the GC box and TATA box-like elements on the transcriptional activity of p*Tktl1-56*. The sequences GGGCGG (GC box) and TTTTAA (TATA box like) were mutated to GGAAAG and TTGCGA, respectively. Plasmids (500 ng/µl) were microinjected into the male pronuclei of one-cell-stage embryos. Experiments were performed three times and the results are presented as means ± SEM. Asterisks indicate significant differences (Student's *t*-test; *P*<0.05).

To find the element(s) responsible for the transcriptional activity in the upstream 56 bp of the TSS in *Tktl1* gene, we explored the transcription factor binding sites (TFBS) in this region and found a GC box (GGGCGG) at −50 to −45 bp from TSS (Fig. 5A). GC box is known as an element that regulates not only the efficiency of gene expression, but also determines the locus of transcription start site [17]. Furthermore, we also found a TATA box like element (TTTTAA) located in −30 to −25 bp region from TSS (Fig. 5A). Although this element has the sequence which is 2 base nucleotides different from TATA box consensus sequence, it was reported that only 2 base nucleotides difference showed no apparent effect on the affinity of transcription factors [18], [19]. To examine whether or not these GC box and TATA box like element is important for *Tktl1* transcription, p*Tktl1-56* was mutated on these elements and examined for the transcriptional activity. The results showed that a mutation on either GC box or TATA box like element markedly reduced the transcriptional activity of p*Tktl1-56* (Fig. 5B), suggesting that the GC box and TATA box like element is essential for the *Tktl1* transcription in the 1-cell stage embryos.

### p*Tktl1-56* did not show transcriptional activity in the 2-cell stage embryos

**Figure 6:**
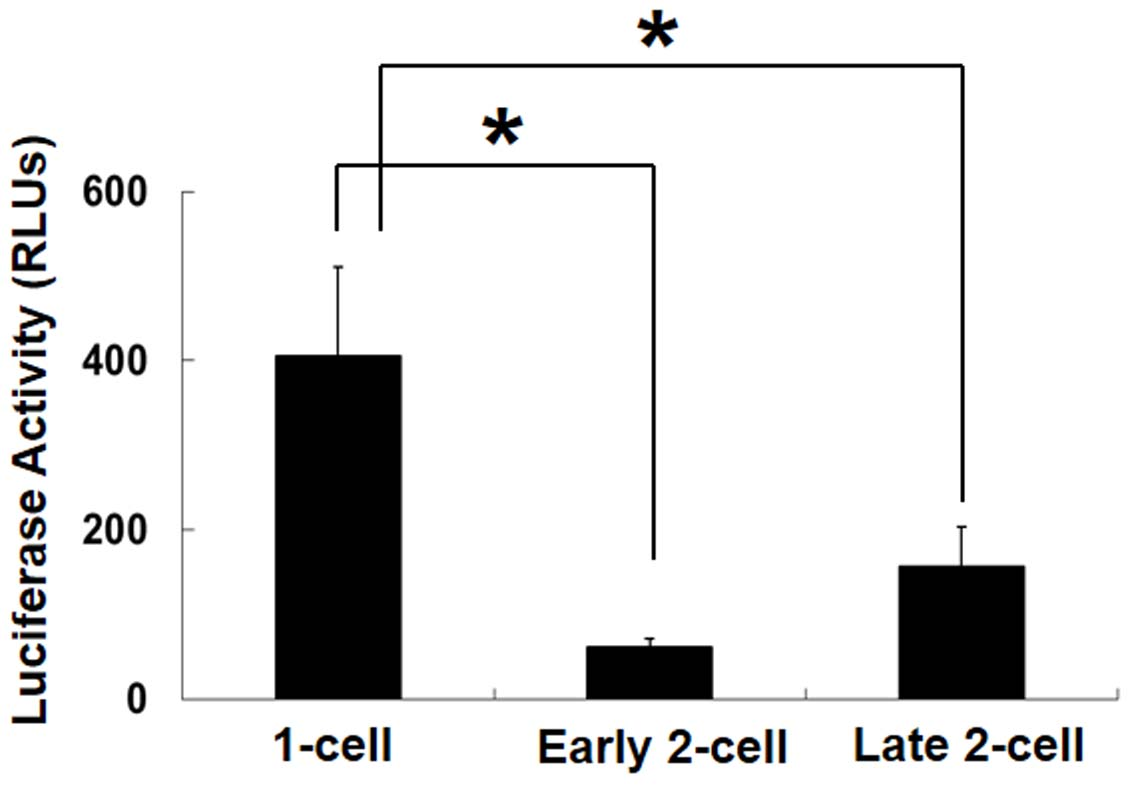
Transcriptional activity of the 56*Tktl1* in one- and two-cell-stage embryos. Plasmids (200 ng/µl) were microinjected into the male pronuclei of one-cell-stage embryos and the nuclei of early and late two-cell-stage embryos. Microinjection was performed at 9, 18 and 27 h after insemination in one-cell and early and late two-cell-stage embryos, respectively. Experiments were performed four times and the results are presented as means ± SEM. Asterisks indicate significant differences (Student's *t*-test; *P*<0.05).

Finally, in order to know whether or not the transcription induced by the upstream 56 bp is a specific phenomenon in 1-cell stage embryos, we examined the transcriptional activity of p*Tktl1-56* in the early and late 2-cell stage embryos. As the results, only a background level of the activity was observed in both stages of the embryos (Fig. 6), although the transcriptional activity of p*Tktl1-2467+25* was much higher in the 2-cell stage embryos than 1-cell stage ones (Fig. 2). These results suggested that the expression of *Tktl1* can be induced only by a core promoter in the 1-cell stage embryos but it cannot be and requires the proximal promoter after cleaved to the 2-cell stage embryos.

## Discussion

In the present study, we investigated the regulation of gene expression in the 1-cell stage embryos by using reporter gene assay. In this assay, we analyzed the transcriptional activity of the upstream region of *Tktl1* gene which is expressed at 1-cell stage. We successfully detected the luciferase activity in the 1-cell stage embryos which had not been treated with any cell cycle inhibitor like an aphidicolin. Therefore, we suppose that our results would well reflect the mechanism regulating the expression of endogenous genes at the 1-cell stage.

In the reporter gene assay using p*Tktl1-2467+25*, the transcriptional activity was higher in the early 2-cell stage than the late one (Fig. 2) and the inhibition of the second round DNA replication greatly increased the activity at the late 2-cell stage (Fig. 3). These results are consistent with those of previous studies suggesting that the embryos are in the transcriptionally permissive state at the 1-cell and early 2-cell stages and that the repressive state develops during 2-cell stage by the mechanism coupling to DNA replication [9], [10], [12], [20]. Therefore, the chromatin structure seems to be loosened in the embryos at 1-cell stage and become tight during DNA replication at 2-cell stage, which leads to the repression of some genes which had been expressed during 1-cell and early 2-cell stages. This hypothesis is supported by the results of previous reports that the treatment of 2-cell embryos with trapoxin, an inhibitor of histone deacetylase, which makes the chromatin loosened, prevented *Eif1a* and the transcription-requiring complex [21] from their repression during the 2-cell stage [20], [22], and the similar results were observed when the second round of DNA replication was inhibited with aphidicolin.

From the results of deletion analysis in the reporter gene assay, we found that only the 56 bp upstream region from the TSS of *Tktl1* was able to raise the basal transcription (Fig. 4). Mutation analysis of p*Tktl1-56* revealed that TATA box and GC box are essential for the transcriptional activity on the 56 bp upstream region (Fig. 5). TATA box, which is present in 30% of human genes on their core promoter regions [23], functions to determine the TSS correctly. Immediately above the TATA box (−38 to −31 bp) on *Tktl1* promoter, we also found TFIIB recognition element (BRE) which is known to work in a coordinated manner with TATA box to establish the fundamental transcription complex on the core promoters [24]. GC box, which is often found in vicinity to the TATA box, is known as a regulation element to determine the transcriptional efficiency and regulate TSS utilization in TATA-less promoters [17]. There are many transcription factors bound to GC box. It has been reported that one of those factors, SP1 was detected in the nucleus at a low level in fully-grown oocyte but its level increased during the 1- and 2-cell stages after fertilization [25]. It was also reported that suppression of *Sp1* abolished the *Hsp70.1* transgene expression in the 2-cell stage embryos [15]. Therefore, *Tktl1* may also be regulated by SP1 through GC box in the core promoter region in the 1-cell stage embryos. In addition, there is another GC box in the upstream of the 56 bp core promoter of *Tktl1* (−64 to −59 bp from the TSS). This second GC box may enhance the basal transcriptional activity of 56 bp core promoter, since promoters are more active when they have multiple GC boxes than a single one [17]. Indeed, the transcriptional activity was higher in p*Tktl1-227+25* than p*Tktl1-56+25* (Fig. 4A).

It seems to be a specific phenomenon in the 1-cell stage embryos that transcription was induced only by a core promoter region, because it was not observed in either of the early or late 2-cell stage embryos (Fig. 6). These results suggest that the mechanism regulating gene expression is different between the 1-cell and 2-cell stage embryos. Previous studies suggested that the transition from the transcriptionally permissive state to the repressed one occurs during 2-cell stage by the mechanism coupled to DNA replication. Consistent with the results of these studies that the transcriptional activity of p*Tktl1-2467+25* was high at the early 2-cell stage but it decreased by 80% at the late one (Fig. 2), and that this decrease in the transcriptional activity was prevented by inhibiting the DNA replication (Fig. 3). However, the transcriptional activity of p*Tktl1-56* activity had been already repressed at the early 2-cell stage (Fig. 6). Therefore, another mechanism which is different from DNA replication coupled mechanism may contribute this repression occurring during transition from the 1- to 2-cell stage. This mechanism may be coupled to the first mitosis, since some reports suggested that the chromatin structure is changed during the first mitosis. DNase sensitivity was relatively high in the 1-cell stage embryos but it decreased after cleaved into the 2-cell embryos [26]. Although the transcriptional activity is higher in the paternal genome than the maternal one in the 1-cell stage embryos [2], [3], [7], it was almost same in both parental genomes which had been kept in the separate positions during the first mitosis, and was allowed to exit the mitotic phase and form their own nuclei by the treatment with 6-demethylaminopurine [27]. These results suggest that some changes in the chromatin structure have already occurred during the first mitosis before the second round of DNA replication. Taken together, the repressive chromatin seems to be established after fertilization by the two steps which occurs during transition from the 1- to 2-cell stage by the mechanism coupled to the first mitosis and then during 2-cell stage by the one coupled to the second DNA replication.
